# P-167. New epidemiological routes of Coccidioidomycosis in Mexico – the extension of this pathogen to new areas

**DOI:** 10.1093/ofid/ofae631.372

**Published:** 2025-01-29

**Authors:** Eduardo Romo Leija, Carlos Eduardo Maldonado Barrientos, Mercedes Aranda-Audelo, Dora Edith Corzo Leon, Sebastian Rodriguez Llamazares, Alejandra Hernandez Teran, Fernando Rosalio Morales Villarreal, Gabriel Palma Cortes, Victor A Hernandez-Hernandez, Carlos Flores Nunez, Eduardo Becerril-Vargas, Victor Hugo Ahumada Topete, Marco Villanueva Reza

**Affiliations:** INER, MEXICO CITY, Distrito Federal, Mexico; INER, MEXICO CITY, Distrito Federal, Mexico; Hospital General Dr. Manuel Gea González, Mexico City, Distrito Federal, Mexico; Medical Research council Centre for Medical Mycology at the University of Exeter Geoffrey Pope Building, Exeter, England, United Kingdom; Queen's University, Kingston, Ontario, Canada; UNAM, Mexico, Distrito Federal, Mexico; INER, MEXICO CITY, Distrito Federal, Mexico; INER, MEXICO CITY, Distrito Federal, Mexico; INER, MEXICO CITY, Distrito Federal, Mexico; INER, MEXICO CITY, Distrito Federal, Mexico; Instituto Nacional de Enfermedades Respiratorias Ismael Cosío Villegas, Mexico City, Distrito Federal, Mexico; INER, MEXICO CITY, Distrito Federal, Mexico; INSTITUTO NACIONAL ENFERMEDADES RESPIRATORIAS, MEXICO CITY, Distrito Federal, Mexico

## Abstract

**Background:**

Due to specific growing conditions, Coccidioidomycosis is a fungal disease typically found in northern Mexico, near California or Arizona. However, due to climate change, there has been an increase in cases in non-endemic areas. In this study, we describe areas where cases of Coccidioidomycosis have been reported, which were previously not known to have this disease.

Image of a map of Mexico that highlights both high-risk and low-risk areas.

**Figure:**
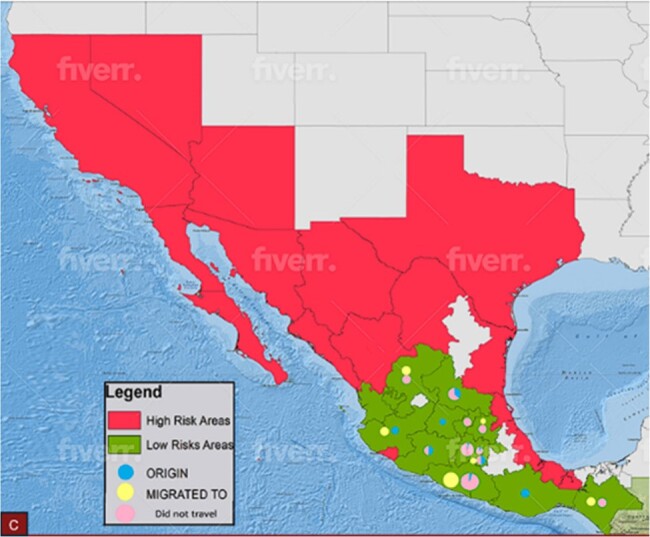

Dots represent origin and migration. Blue circles represent areas where patients lived while yellow represents areas where people migrated. The reason for migration was not studied. Pink circles represent those without travelling.

**Methods:**

We developed a registry of Coccidioidomycosis cases, which collects data on sociodemographic, travel, and clinical conditions, including tomography, pathology, and outcomes. The patients were categorized into groups based on migration and geographical living area, and we collected data from physical or electronic health records.

**Results:**

Between 1991 and 2023, we diagnosed 122 patients with coccidioidomycosis. The most common comorbidities were diabetes mellitus (41%) and overweight (24%). Forty-eight patients (39%) living in endemic areas had high-risk working conditions, such as construction, archaeology, and topography. Diagnosis was made using culture in 79.5% of cases, serology in 54%, and biopsy in 51%. CT scans showed predominant nodular (77%) and cavitary lesions (61%). Surprisingly, 46.7% of patients had no risk factors, such as travelling or living in endemic areas. 29.5% of patients had a history of migration as a risk factor to a high-risk area. The average time from symptom onset to diagnosis was 150 days (IC95 61-518 days). The patients were divided into four groups based on their risk factors. 8 (6.5%) lived and travelled in high-risk areas, 19 (15.5%) lived in high-risk areas without migration, 36 (29.5%) lived in low-risk, travelling to high-risk, and 57(46.7%) lived in low-risk and did not travel.

**Conclusion:**

This division through risk factors highlights areas not known to be at risk, with patients without a history of travelling presenting with Coccidioidomycosis. We estimate an increasing number of fungal infections due to climate change, characterized by increased drought in some areas. As with other diseases, diagnosing Coccidioidomycosis outside of endemic areas should raise awareness of its expansion, and healthcare workers should consider it as a possible differential diagnosis. It is crucial to have this area known to consider resources for treatment.

**Disclosures:**

**Carlos Flores Nunez, PHD**, Pfizer: Grant/Research Support

